# A cross‐sectional study of caesarean deliveries performed by medical doctors and associate clinicians in nine district and regional hospitals in Sierra Leone using the Robson Ten Group Classification System

**DOI:** 10.1002/ijgo.71072

**Published:** 2026-05-30

**Authors:** Josien Westendorp, Rugiatu Jalloh, Sarrah Sheriff, Jenny Wussow, Guro Stamland, Sigrid‐Anne Bjorøy, Francis Moses, Marcus J. Rijken, Risa Hoffmann‐Lonnee, Håkon A. Bolkan, Alex J. van Duinen

**Affiliations:** ^1^ Institute of Public Health and Nursing The Norwegian University of Science and Technology Trondheim Norway; ^2^ CapaCare Masanga Sierra Leone; ^3^ College of Medicine and Allied Health Sciences University of Sierra Leone Freetown Sierra Leone; ^4^ Masanga Medical Research Unit Masanga Sierra Leone; ^5^ Global Health Section, Department of Public Health University of Copenhagen Copenhagen Denmark; ^6^ Faculty of Medicine and Health Sciences The Norwegian University of Science and Technology Trondheim Norway; ^7^ Government of Sierra Leone: Ministry of Health Director of Reproductive Health Freetown Western Area Sierra Leone; ^8^ The Julius Center for Health Sciences and Primary Care Utrecht The Netherlands; ^9^ Netherlands Cancer Institute Amsterdam The Netherlands; ^10^ Department of Obstetrics and Gynecology, St. Olavs Hospital HF Trondheim University Hospital Trondheim Norway; ^11^ Department of Surgery, St. Olavs Hospital HF Trondheim University Hospital Trondheim Norway

**Keywords:** caesarean deliveries, perinatal mortality, Robson Ten Group Classification System, Sierra Leone, task‐sharing

## Abstract

**Objective:**

Maternal and neonatal mortality remain high in Sierra Leone despite significant decline in the last decade. The aim of the present study was to evaluate obstetric care delivery using the Robson Ten Group Classification System (TGCS), focusing on caesarean delivery (CD) and task‐sharing.

**Methods:**

This cross‐sectional study analyzed maternity and surgical registry data from nine governmental hospitals, collected over 4 months in 2021 or 2022. Variables included mode and outcome of delivery, TGCS parameters, and provider cadre: associate clinician (AC) or medical doctor (MD).

**Results:**

Of 5748 women included in the study, 3806 (66.2%) delivered vaginally, 156 (2.7%) by assisted vaginal delivery and 1786 (31.1%) by CD. The largest TGCS groups were low risk primipara (28.8%) and multipara (47.0%). In the low risk primipara group, 450/1656 (27.2%) underwent CD; in the low risk multipara group, 604/2702 (22.4%) underwent CD. MDs performed 876 (49.1%) of the total CDs and ACs 830 (46.5%). ACs performed more CDs with the indication of twin pregnancy, previous CD, or malpresentation, whereas MDs performed more with the indication of breech, uterine rupture, and fetal distress.

**Conclusion:**

Facility‐based CD rates in Sierra Leone are high, particularly among low risk primi‐ and multipara‐Robson groups, while assisted vaginal delivery rates remain low. Nearly half of all CDs are performed by ACs, highlighting the importance of task‐sharing in this context. Further in‐depth analysis is warranted to understand the factors influencing CD decision making and the collaborative processes between ACs and MDs to improve maternal and neonatal outcomes.

## 1 INTRODUCTION

The decision to perform a caesarean delivery (CD) is often complex and dependent on the context.^1^, ^2^ In many parts of the world this life‐saving procedure remains unjustifiably underused, often due to inadequate access.^3^ In other parts, an increasing number of women are subjected to surgical births in operating theaters, even when a normal or assisted vaginal delivery would be safer and more beneficial.^4^, ^5^ In low‐income contexts these CDs without medical indications are draining already scarce resources.^6^ Importantly, without a medically necessary reason to perform a CD, women and their offspring are put at greater risk of poor outcomes in subsequent pregnancies, now and for the years to come.^5^, ^7^, ^8^, ^9^ Clinical decision making for a CD, particularly in the absence of a clear‐cut medical indication, is shaped by clinicians' beliefs, fear of adverse outcomes, and systemic factors embedded within the healthcare context.^10^

The WHO recommends Robson's Ten Group Classification System (TGCS) to enhance the understanding of decision making processes related to CDs.^4^ By enabling standardized analysis and comparison of caesarean section rates, TGCS facilitates reflection on clinical practice within hospitals, which can be extended to national and global levels to support shared learning and improved care.^2^, ^11^ By analyzing and discussing TGCS data, obstetric care provider teams obtain more information and insights into their specific obstetric practices.^12^ In low‐resource settings, the collection of these data can be challenging, and formal discussions around them may be limited.

A population CD rate of 10% has been proven to significantly improve outcomes for mothers and babies; however, in Sierra Leone the population CD rate is only 4.4%.^13^, ^14^, ^15^ In many districts CD rates are even lower, indicating that medically justifiable CDs are not performed.^16^, ^17^ This is reflected in the fact that maternal and neonatal mortality and morbidity are still high despite significant improvements.^16^, ^18^, ^19^, ^20^

Several interventions have resulted in a pivotal reduction in maternal mortality ratio from 1680 women per 100 000 livebirths in 2000, to 354 per 100 000 in 2023.^20^ Among these interventions are the Free Health Care Initiative, increasing skilled birth attendance by training midwives and the introduction of a transportation ambulance system.^21^, ^22^, ^23^

One other intervention, aiming to improve access to CDs through task‐sharing, was the implementation of a training program for community health officers and non‐specialized medical doctors (MDs), implemented by the Ministry of Health and the Non‐Governmental Organization CapaCare.^24^ This program established in 2011, is based on experience from East Africa where a large proportion of CDs are carried out by associate clinicians (ACs).^25^, ^26^ The 3‐year training program trains and qualifies graduates, known as ACs, to manage surgical emergencies, such as CDs.^24^ In 2023, 57.7% of CDs were carried out by ACs.^27^ A prospective cohort study from Sierra Leone concluded that CDs performed by ACs were non‐inferior compared with those undertaken by MDs comparing maternal mortality.^28^

The decision making for CDs and the collaborative processes between ACs and MDs remain largely unexplored. MDs generally perform more complex procedures, often outside office hours or as emergency operations, while in many cases both cadres are not present simultaneously.^28^ Assignment of a CD to an MD or AC is not governed by formal protocols but depends primarily on provider availability and experience.

The primary aim of this study was to investigate the rates, indications, and perinatal outcomes of deliveries classified according to the TGCS in nine district and regional government hospitals in Sierra Leone. It also aimed to examine how CDs and their indications differed between MDs and ACs across TGCS groups.

## 2 MATERIALS AND METHODS

### 2.1 Study setting

Sierra Leone is a country in West‐Africa with a population of 8.5 million in 2023.^29^ The fertility rate dropped from 6.4 total births per woman in 2000 to 3.8 in 2023.^29^ Of all deliveries, 83.4% take place in a health facility and 86.9% are assisted by a skilled health professional.^17^ The population CD rate is 4.4%, and access to CD are unevenly distributed across the country.^17^, ^30^

The combined surgical, anesthetic and obstetric specialist workforce is 0.13 per 100 000 population, which is more than 100 times less than the benchmark proposed by the Lancet Commission on Global Surgery.^31^, ^32^ Most specialists work in the Western Area where the capital Freetown is located and many of the specialists also have administrative duties in addition to clinical work.

The public healthcare system in Sierra Leone is structured into three levels: primary healthcare units, district hospitals and referral hospitals. Most deliveries take place in primary healthcare units, and when complications arise, women are referred to the next level. In the eight district hospitals more specialized services are offered, such as CDs and inpatient care. The highest level of healthcare is provided at the four regional hospitals and at the national referral hospital, the country's only tertiary hospital at the time of this study, located in Freetown.

### 2.2 Study design

This was a cross‐sectional study with a 4‐month sample of obstetric activity in 2021 (4 hospitals) or 2022 (5 hospitals) in the nine most productive district and regional governmental hospitals in Sierra Leone. All governmental district and regional hospitals with a minimum of 200 CDs per year based on activity in 2017, where both ACs and MDs provided obstetrical surgical care during the study period, were eligible for inclusion in the study.^33^

### 2.3 Data collection

Data from 2021 were collected by two Norwegian fifth year medical students (GS and SAB) in 2022, and data from 2022 were collected by a Sierra Leonean Senior Registered Nurse (SS), a German graduate student in global health (JWu) together with the primary investigator (JW) in 2023. All data collectors were trained and (in)directly supervised by the primary investigator (JW). A 4‐month sample was selected (January, May, September, and October), evenly distributed throughout the year to account for seasonal variety.

If no data were available for more than 2 days of the complete month, the following complete full month was used as a substitute. The primary sources for the data were the handwritten maternity and delivery register books provided by the Ministry of Health to all governmental facilities. For all women who underwent a CD, the information was verified in the Ministry of Health operation theater registry. Photographs of these registries were taken in the facilities and stored on a protected research hard‐disk and entered in a non‐identifiable Excel database.

### 2.4 Participants inclusion and exclusion criteria

All deliveries with a gestational age of 24 weeks or more were included. Deliveries of offspring weighing less than 500 g were excluded. A live birth was defined as the complete expulsion or extraction of a product of conception, regardless of gestational age, that after separation breathes or shows any sign of life (heartbeat, umbilical cord pulsation, or voluntary movement). A stillbirth was defined as fetal death before complete expulsion or extraction, with no signs of life after birth (no breathing, heartbeat, umbilical cord pulsation, or movement). Information on the timing of stillbirths (ante‐ or intrapartum) was not available.^34^ All deliveries were retrospectively classified according to the WHO standard Robson definitions, based on variables recorded in the maternity registries: parity, previous CD, onset of labor (spontaneous, induced, or CD before labor), number of fetuses (singleton or multiple), gestational age (preterm <37 weeks or term ≥37 weeks), and fetal lie and presentation (cephalic, breech, or transverse).^35^ Each woman was assigned to one mutually exclusive Robson group. Robson group 7 includes multiparous women with a singleton breech pregnancy, including those with a previous CD.

As gestational age was not always available or reliable due to limited access to diagnostic ultrasound, birth weight was used as a proxy. Neonates with birth weight < 2500 g were classified as preterm, and those ≥2500 g as term. For neonates weighing 2000–2499 g with documented gestational age, those <37 weeks were classified as preterm and those ≥37 weeks as term. Additionally, data on maternal and neonatal outcomes, mode of delivery, and whether the CD was performed by an MD or AC were recorded. Indications for CD, as documented in the theater registries, were classified according to Anderson and Althabe.^36^, ^37^

### 2.5 Handling of missing data

After the initial data collection, the same team conducted an additional visit to retrieve missing information from patient files. Files were consulted for women with missing data on Robson TGCS or mode of delivery. Women with unknown mode of delivery were excluded. Deliveries with incomplete data for Robson TGCS were not excluded but reported as missing.^35^

### 2.6 Statistical analysis

Anonymized data was entered into a Microsoft Excel version 16.602022 database (Microsoft Corporation, Redmond, Washington, USA). Statistical analyses were performed with Stata/MP version 19.5 (StataCorp LLC, College Station, Texas, USA).

Baseline characteristics are presented as counts (percentages) for categorical variables and as means with standard deviations (SDs) for continuous variables. A Fisher exact test was used for testing categorical data. The odds for CDs performed by AC were compared as ratio with *P* value for each of the TGCS groups and CD indications compared with the rest of the cohort. A *P* value of less than 0.05 was considered statistically significant.

### 2.7 Ethical considerations and informed consent

Ethical clearance was given by the Sierra Leone Ethics and Scientific Review committee (024/04/2023) and the Masanga Medical Research Committee (MMRU‐SRC‐01‐2023). The Regional Committee for Medical and Health related Research Ethics Mid Norway (REK Midt) considered this study as a quality improvement project and therefore not requiring ethical approval from Norway. Informed consent was obtained from the hospital leadership of each participating facility before the start of data collection. Data were anonymized prior to being accessed by the study authors. Informed consent from patients was therefore not required.

## 3 RESULTS

Between February and April 2022 and between May and June 2023, a 4‐month data sample of the previous calendar year was collected in respectively four and five governmental hospitals (Figure 1). Of the nine hospitals, four were regional hospitals and five were district hospitals (Table 1; Figure 2). For 4 months, data was incomplete and was therefore replaced by the following complete month. In total, 5796 women were assessed for eligibility. For 114 women a second round of data collection from the patient files was undertaken to review the data. In total, 48 (0.7%) women were excluded, and 5748 (99.3%) were included for the analysis.

**FIGURE 1:**
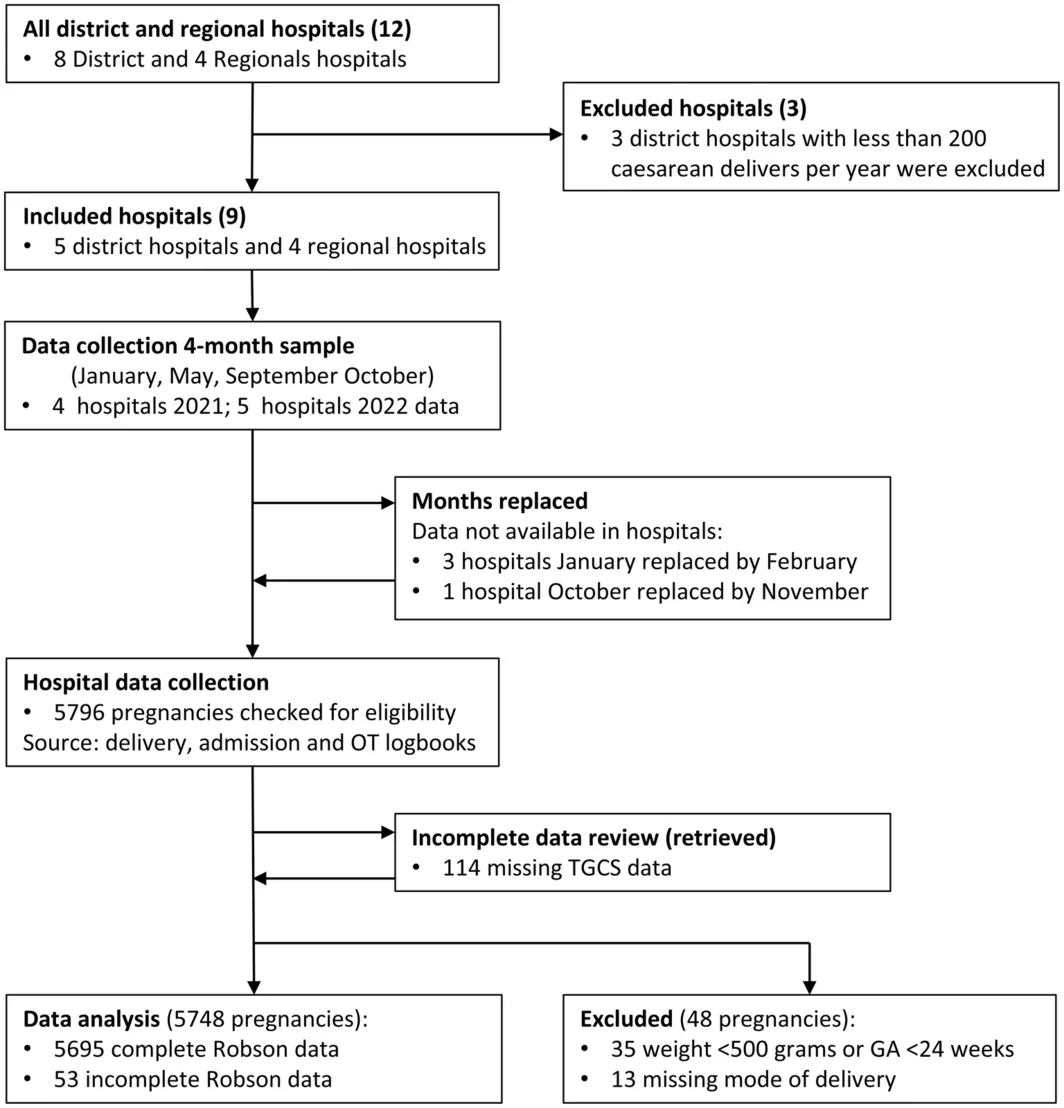
Study flow chart.

**TABLE 1:** Hospital characteristics.

Hospital	Deliveries^a^			Number of CD by		
	NVD	AVD	CD	MD	AC	Other/unknown
District hospital 1^b^	368 (71.5%)	3 (0.6%)	144 (28.0%)	98 (68.1%)	40 (27.8%)	6 (4.2%)
District hospital 2^c^	279 (68.6%)	22 (5.4%)	106 (26.0%)	5 (4.7%)	90 (84.9%)	11 (10.4%)
District hospital 3^b^	401 (51.4%)	6 (0.8%)	373 (47.8%)	321 (86.1%)	30 (8.0%)	22 (5.9%)
District hospital 4^b^	460 (76.4%)	23 (3.8%)	119 (19.8%)	51 (42.9%)	61 (51.3%)	7 (5.9%)
District hospital 5^c^	142 (40.5%)	28 (8.0%)	181 (51.6%)	169 (93.4%)	10 (5.5%)	2 (1.1%)
Regional hospital 1^c^	788 (70.9%)	21 (1.9%)	302 (27.2%)	7 (2.3%)	290 (96.0%)	5 (1.7%)
Regional hospital 2^c^	547 (65.3%)	33 (4.0%)	258 (30.8%)	3 (1.2%)	237 (91.9%)	18 (7.0%)
Regional hospital 3^b^	677 (73.7%)	9 (1.0%)	233 (25.4%)	182 (78.1%)	46 (19.7%)	5 (2.2%)
Regional hospital 4^c^	144 (64.0%)	11 (4.9%)	70 (31.1%)	40 (57.1%)	26 (37.1%)	4 (5.7%)
Total	3806 (66.2%)	156 (2.7%)	1786 (31.1%)	876 (49.1%)	830 (46.5%)	80 (4.5%)

Abbreviations: AC, associate clinician; AVD, assisted vaginal deliveries; CD, caesarean delivery; MD, medical doctor; NVD, normal vaginal delivery.

^a^ Four‐month sample of deliveries, percentages show proportion of all deliveries.

^b^ Data collection in 2021.

^c^ Data collection in 2022.

**FIGURE 2:**
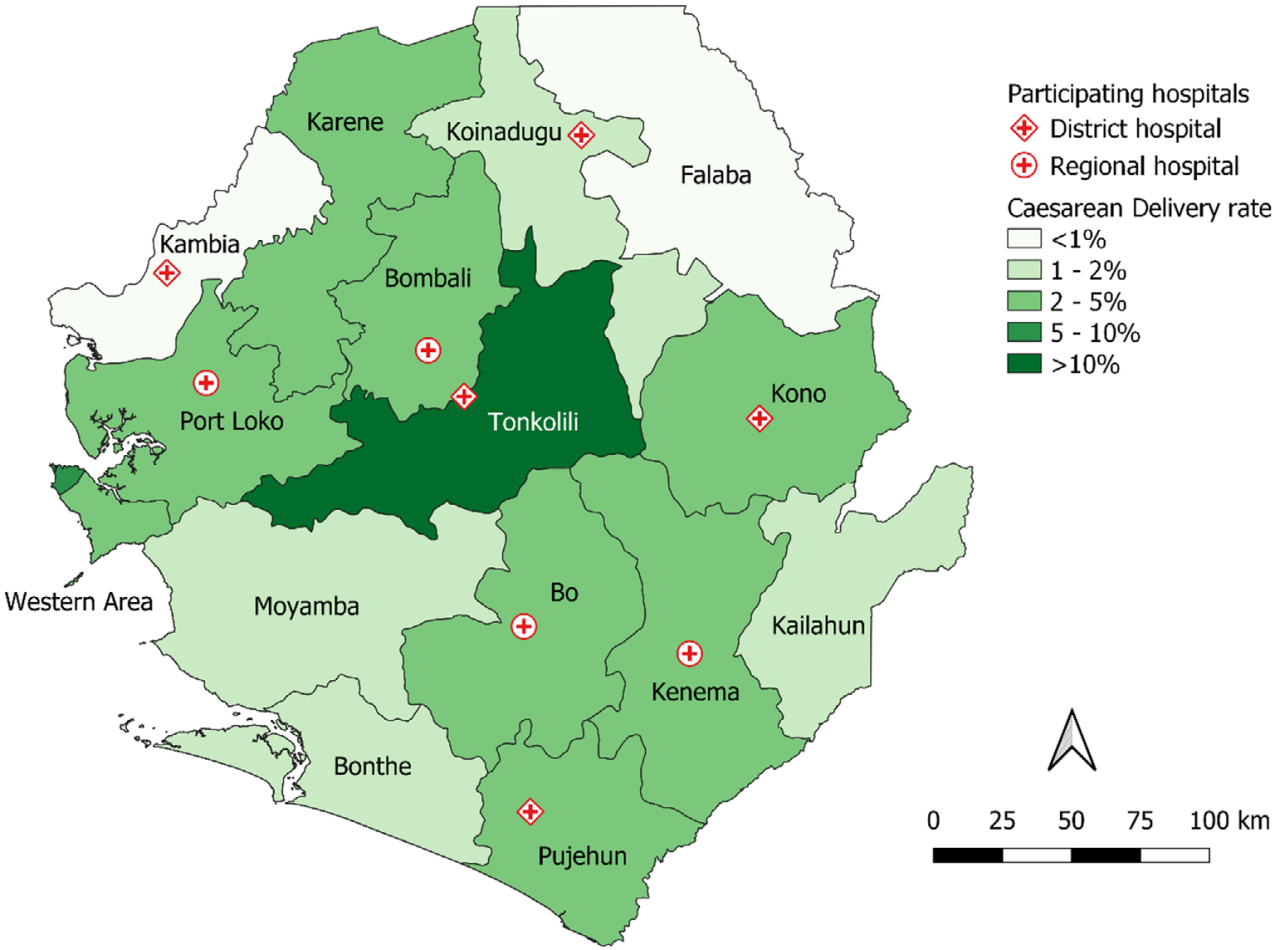
Participating hospitals by category and population caesarean delivery rates. Participating district and regional hospitals and population Caesarean Delivery rates from: Sierra Leone Demographic and Health Survey 2019 (Statistics Sierra Leone (Stats SL) and ICF. 2020, Freetown, Sierra Leone, and Rockville, Maryland, USA).

The mean age of the pregnant women was 24.4 years (SD 5.9) with a mean gestational age of 37.3 weeks (SD 2.5) (Table 2). Of the 5748 pregnancies, 301 (5.2%) were twin pregnancies and one was a triplet (Table 2). For 19 women (0.3%) a maternal death was recorded in the registers (Table 2). More than half of the women (*n* = 3143; 54.7%) were multipara (para 1 to 4), followed by 2037 (35.4%) nullipara, 518 (9.0%) grand multipara (para 5 or more) and 50 (0.9%) missing parity (Table 2). In total, 364 (6.3%) of the women had one previous CD and 27 (0.5%) had two or more previous CDs (Table 2). The nine facilities had a combined CD rate of 31.1%, ranging between 19.8% and 51.6%, the overall assisted vaginal delivery (AVD) rate was 2.7%, ranging between 0.6% and 8.0% (Table 1).

**TABLE 2:** Pregnancy characteristics.

Indicator	All pregnancies
Age women, years^a^	24.4 (5.9)
Gestational age, weeks^a^	37.3 (2.5)
Single/multiple pregnancy, *n* (%)	
Single pregnancy	5446 (94.8%)
Multiple pregnancy	301 (5.2%)
Triplet pregnancy	1 (0.0%)
Parity, *n* (%)	
Nullipara (p0)	2037 (35.4%)
Multipara (p1–4)	3143 (54.7%)
Grand multipara (≥p5)	518 (9.0%)
Missing	50 (0.9%)
Previous CD, *n* (%)	
None	5350 (93.1%)
1	364 (6.3%)
2 or more	27 (0.5%)
Missing	6 (0.1%)
Position^b^	
Cephalic	5102 (88.8%)
Breech	227 (4.0%)
Transverse	54 (0.9%)
Missing	365 (6.4%)
Perinatal outcome, *n* (%)	
Alive	5502 (90.9%)
Perinatal death	462 (7.6%)
Missing	87 (1.4%)
Maternal outcome, *n* (%)	
Alive	4477 (77.9%)
Death	19 (0.3%)
Missing	1252 (21.8%)

Abbreviations: CD, caesarean delivery; SD, standard deviation.

^a^ Values are mean (SD).

^b^ In case of multiple pregnancy, position of the first baby.

From the 5748 women included in the study, for 53 (0.9%) there was insufficient data to be classified into the TGCS groups (Table 3 and Table S1). The two largest Robson groups were group 3 (multiparous women, without a previous CD; *n* = 2702; 47.0%) and group 1 (nulliparous women, single cephalic, ≥37 weeks, spontaneous labor; *n* = 1656; 28.8%) (Table 3). Considerable variation was observed in the relative size of the largest Robson groups across hospitals: group 1 (nulliparous women, single cephalic, ≥37 weeks, spontaneous labor) ranged from 16.4% to 35.6%, and group 3 (multiparous women without a previous CD, single cephalic, ≥37 weeks, in spontaneous labor) from 42.5% to 54.8% (Figure S1). Robson group 1 and 3 contributed to most of the CDs (*n* = 450 (relative contribution of group CD to overall CD was 25.2%) and *n* = 604 (relative contribution of group CD to overall CD was 33.8%), respectively) (Table 3 and Table S1).

**TABLE 3:** Mode of delivery and caesarean delivery provided by TGCS.

Robson group		Total^a^	Mode of delivery			Contribution CD by			ACOR (95% CI)^b^
			NVD	AVD	CD	MD	AC	Unknown	*P* value
1	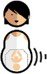	1656 (28.8%)	1125 (67.9%)	81 (4.9%)	450 (27.2%)	224 (49.8%)	216 (48.0%)	10 (2.2%)	1.09 (0.88–1.34) *P* = 0.453
2	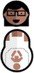	29 (0.5%)	5 (17.2%)	2 (6.9%)	22 (75.9%)	14 (63.6%)	7 (31.8%)	1 (4.5%)	0.53 (0.22–1.32) *P* = 0.172
3	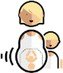	2702 (47.0%)	2049 (75.8%)	49 (1.8%)	604 (22.4%)	330 (54.6%)	252 (41.7%)	22 (3.6%)	0.75 (0.61–0.91) ***P* = 0.004**
4	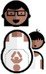	71 (1.2%)	8 (11.3%)	0 (0.0%)	63 (88.7%)	46 (73.0%)	13 (20.6%)	4 (6.3%)	0.29 (0.16–0.53) ***P* < 0.001**
5	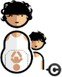	291 (5.1%)	93 (32.0%)	8 (2.7%)	190 (65.3%)	69 (36.3%)	113 (59.5%)	8 (4.2%)	1.80 (1.32–2.44) ***P* < 0.001**
6	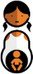	66 (1.2%)	31 (47.0%)	0 (0.0%)	35 (53.0%)	17 (48.6%)	16 (45.7%)	2 (5.7%)	0.97 (0.50–1.90) *P* = 0.928
7	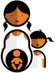	109 (1.9%)	46 (42.2%)	4 (3.7%)	59 (54.1%)	25 (42.4%)	32 (54.2%)	2 (3.4%)	1.38 (0.82–2.32) *P* = 0.226
8	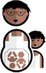	281 (4.9%)	141 (50.2%)	6 (2.1%)	134 (47.7%)	63 (47.0%)	61 (45.5%)	10 (7.5%)	0.96 (0.67–1.37) *P* = 0.819
9	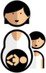	45 (0.8%)	3 (6.7%)	0 (0.0%)	42 (93.3%)	16 (38.1%)	23 (54.8%)	3 (7.1%)	1.41 (0.76–2.60) *P* = 0.278
10	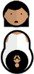	445 (7.7%)	298 (67.0%)	6 (1.3%)	141 (31.7%)	56 (39.7%)	70 (49.6%)	15 (10.6%)	1.15 (0.81–1.62) *P* = 0.432
Missing		53 (0.9%)	7 (13.2%)	0 (0.0%)	46 (86.8%)	16 (34.8%)	27 (58.7%)	3 (6.5%)	1.66 (0.92–3.00) *P* = 0.095
Total		5748 (100.0%)	3806 (66.2%)	156 (2.7%)	1786 (31.1%)	876 (49.1%)	830 (46.5%)	80 (4.5%)	N/A

*Note*: TGCS groups: 1 = nulliparous, single cephalic, ≥37 weeks, spontaneous labor; 2 = nulliparous, single cephalic, ≥37 weeks, induced or CD before labor; 3 = multiparous (excluding previous CD), single cephalic, ≥37 weeks, in spontaneous labor; 4 = multiparous (excluding previous CD), single cephalic, >37 weeks, induced or CD before labor; 5 = previous CD, single cephalic, ≥ 37 weeks; 6 = all nulliparous breeches; 7 = multiparous breeches; 8 = multiple pregnancies; 9 = abnormal lies; 10 = single cephalic, <37 weeks. *P* < 0.05 were made bold to underline significance.

Abbreviations: AC, associate clinician; ACOR, associate clinician odds ratio; AVD, assisted vaginal deliveries; CD, caesarean delivery; CI, confidence interval; MD, medical doctor; N/A, non‐applicable; NVD, normal vaginal delivery; ref., reference group; TGCS, Robson's Ten Group Classification System.

^a^ Percentages show proportion of all deliveries.

^b^ ACOR is the ratio between the odds that a CD is performed by a AC for the specific TGCS group the rest of the cohort.

The CD rate was the highest in group 9 (abnormal lies; 93.3%, relative contribution of group CD to overall CD was 2.4%) and group 4 (multiparous [excluding previous CD], single cephalic, >37 weeks, induced or CD before labor; 88.7%, relative contribution of group CD to overall CD was 3.5%) followed by group 2 (nulliparous, single cephalic, ≥37 weeks, induced or CD before labor; 75.9%, relative contribution of group CD to overall CD was 1.2%) and group 5 (previous CD, single cephalic, ≥ 37 weeks; 65.3%, relative contribution of group CD to overall CD was 10.6%) (Table 3 and Table S1). Slightly more CDs were performed by MDs (*n* = 876; 49.1%) compared with ACs (*n* = 830; 46.5%) (Tables 3 and 4). The odds of CD performed by an AC were significantly lower in group 4 (multiparous women without a previous CD, single cephalic, >37 weeks, induced or CD before labor) (OR 0.29; *P* < 0.001) and group 3 (multiparous women without a previous CD, single cephalic, ≥37 weeks, in spontaneous labor) (OR 0.75; *P* = 0.004) and higher in group 5 (women with a previous CD, single cephalic, ≥ 37 weeks) (OR 1.80; *P* < 0.001).

**TABLE 4:** Indication for CD by provider.

Indication	Total CD	CD performed by			ACOR (95% CI)^a^ ^s^	
		MD	AC	unknown	OR	*P* value
CPD including obstructed labor	496 (27.8%)	255 (51.4%)	227 (45.8%)	14 (2.8%)	0.96 (0.78–1.18)	0.711
Previous CD	217 (12.2%)	85 (39.2%)	126 (58.1%)	6 (2.8%)	1.70 (1.28–2.27)	**<0.001**
Mechanical/dynamic dystocia	210 (11.8%)	100 (47.6%)	103 (49.1%)	7 (3.3%)	1.12 (0.84–1.50)	0.426
Fetal distress	120 (6.7%)	76 (63.3%)	39 (32.5%)	5 (4.2%)	0.53 (0.36–0.79)	**0.002**
Malpresentation	99 (5.5%)	41 (41.4%)	57 (57.6%)	1 (1.1%)	1.60 (1.07–2.42)	**0.024**
Emergency—placenta abruptio	68 (3.8%)	34 (50.0%)	33 (48.5%)	1 (1.5%)	1.09 (0.67–1.77)	0.729
Emergency—eclampsia	61 (3.4%)	37 (60.7%)	23 (37.7%)	1 (1.6%)	0.69 (0.41–1.17)	0.165
Emergency—placenta previa	48 (2.7%)	23 (47.9%)	25 (52.1%)	0 (0.0%)	1.26 (0.71–2.24)	0.430
Emergency—other	40 (2.2%)	27 (67.5%)	11 (27.5%)	2 (5.0%)	0.43 (0.21–0.86)	**0.018**
Twins	38 (2.1%)	8 (21.1%)	28 (73.7%)	2 (5.3%)	3.30 (1.59–6.84)	**0.001**
Emergency—uterine rupture	36 (2.0%)	27 (75.0%)	9 (25.0%)	0 (0.0%)	0.38 (0.18–0.81)	**0.012**
Breech	32 (1.8%)	24 (75.0%)	7 (21.9%)	1 (3.1%)	0.32 (0.14–0.74)	**0.008**
Failed induction	21 (1.2%)	13 (61.9%)	8 (38.1%)	0 (0.0%)	0.71 (0.29–1.71)	0.441
Other	218 (12.2%)	116 (53.2%)	92 (42.2%)	10 (4.6%)	0.82 (0.62–1.09)	0.178
Missing indication	82 (4.6%)	10 (12.2%)	42 (51.2%)	30 (36.6%)	1.22 (0.78–1.90)	0.378
Total	1786 (100.0%)	876 (46.5%)	830 (49.0%)	80 (4.5%)	N/A	N/A

Abbreviations: AC, associate clinician; ACOR, associate clinician odds ratio; CD, caesarean delivery; CI, confidence interval; CPD, cephalopelvic disproportion; MD, medical doctor. *P* < 0.05 were made bold to underline significance.

^a^ ACOR is the ratio between the odds that a CD is performed by an AC for this indication and the rest of the cohort.

The most frequently reported indication for CD was cephalopelvic disproportion (CPD) including obstructed labor (496; 27.8%), followed by previous CD (217; 12.2%) and mechanical/dynamic dystocia (210; 11.8%) (Table 4). The odds of CDs provided by ACs were higher for CDs performed for the indication of twin pregnancies (OR 3.30; *P* = 0.001), previous CD (OR 1.70; *P* < 0.001) and malpresentation (OR 1.60; *P* = 0.024). On the contrary, the odds of the CD performed by an AC were lower for the indications breech (OR 0.32; *P* = 0.008), uterine rupture (OR 0.38; *P* = 0.012) and fetal distress (OR 0.53; *P* = 0.002).

A total of 6051 babies were recorded, among those 462 (7.6%) were perinatal deaths and for 87 (1.4%) the outcome was unknown. The perinatal mortality rate for babies delivered with normal vaginal delivery (NVD) was 6.8%, compared with 5.6% and 9.5% for babies delivered with AVD and CD, respectively. The highest perinatal mortality for babies delivered by NVD occurred in TGCS group 6 (nulliparous breeches) and group 7 (multiparous breeches), 25.8% and 26.1%, respectively (Figure 3). For babies delivered by CD the highest perinatal mortality was identified in TGCS group 4 (multiparous women without a previous CD, single cephalic, >37 weeks, induced or CD before labor) and group 6 (all nulliparous women with breeches), with 24.6% and 20.0%, respectively. Notably in group 10 (women with a single cephalic, <37 weeks pregnancy) the perinatal mortality was high, above 15%, in both NVD and CD groups. With 180 (39.0%) perinatal deaths, TGCS group 3 (multiparous women without a previous CD, single cephalic, ≥37 weeks, in spontaneous labor) was the largest contributor to the total number of perinatal deaths (see Figure 3).

**FIGURE 3:**
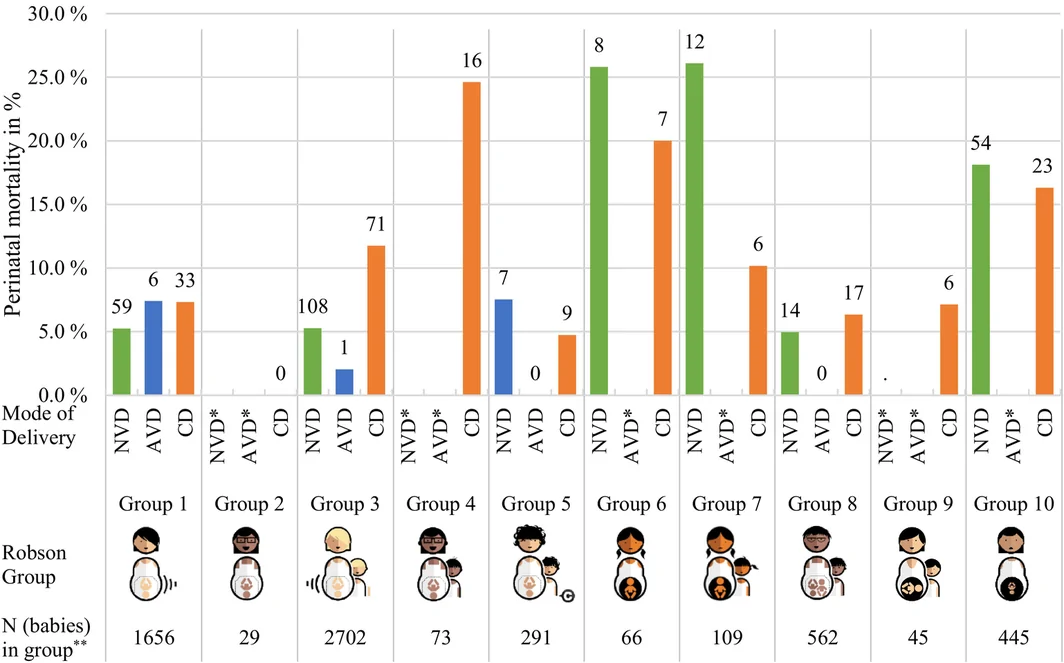
Perinatal mortality by mode of delivery and Robson Ten Group Classification. Perinatal mortality by mode of delivery and Robson Ten‐Group Classification. The numbers on top of the bars are the absolute number of perinatal deaths per subgroup. *Groups with 10 or less participants are not presented in the figure. **Number of babies per group with known outcome. 73 babies were not classified in the Robson groups. AVD, assisted vaginal delivery; CD, caesarean delivery; NVD, normal vaginal delivery. Robson groups: 1 = Nulliparous, single cephalic, ≥37 weeks, spontaneous labour; 2 = Nulliparous, single cephalic, ≥37 weeks, induced or CD before labour; 3 = Multiparous (excluding previous CD), single cephalic, ≥37 weeks, in spontaneous labour; 4 = Multiparous (excluding previous CD), single cephalic, >37 weeks, induced or CD before labour; 5 = Previous CD, single cephalic, ≥ 37 weeks; 6 = All nulliparous breeches; 7 = Multiparous breeches; 8 = Multiple pregnancies; 9 = Abnormal lies; 10 = Single cephalic, <37 weeks.

## 4 DISCUSSION

The present study reports a high overall CD in facility rate of 31.1% while the AVD rate was only 2.7%. The TGCS group contributing most to all CDs was group 3 (multiparous women without a previous CD, single cephalic, ≥37 weeks, in spontaneous labor) with a group CD rate of 33.8%. The most frequent indication for CD in this group was “CPD including obstructed labor” with 27.8%. When comparing ACs with MDs, the odds of performing a CD were significantly higher for ACs in Robson group 5 (women with a previous CD, single cephalic fetus ≥37 weeks). Conversely, the odds were lower for ACs in Robson group 3 (multiparous women without a previous CD, single cephalic, ≥37 weeks, in spontaneous labor) and Robson group 4 (multiparous women without a previous CD, single cephalic, >37 weeks, induced labor or CD before labor). Regarding CD indications: the odds of CDs being performed by ACs (compared with MDs) were higher for twin pregnancies, previous CD, and malpresentation. In contrast, the odds were lower for indications such as breech presentation, uterine rupture, and fetal distress (see Tables 3 and 4). The overall perinatal mortality rate for babies was 7.6%, for babies delivered with NVD this was 6.8%, compared with 5.6% and 9.9% for babies delivered with AVD and CD, respectively (Table 2).

### 4.1 Facility and population CD rates

In a recent publication, the national tertiary referral center in Sierra Leone reported an institutional CD rate of 49.6%, which is even higher than the reported 31.1% CD rate in the district and regional hospitals in this study.^36^ The high facility CD rate in the context of a low population CD rate, can be partly explained by relatively few women delivering in a facility able to provide a CD.^16^, ^17^ The high institutional CD rate can furthermore partly be explained by a high number of referred and already complicated cases upon arrival, resulting in high perinatal and maternal mortality rates, even in the low‐risk TGCS groups 1 (nulliparous women, single cephalic ≥37 weeks, spontaneous labor) and 3 (multiparous women without a previous CD, single cephalic ≥37 weeks, in spontaneous labor), reflecting the limited resources available in the Sierra Leonean healthcare system.^17^, ^36^

### 4.2 Robson groups

Almost half of the women were classified in TGCS group 3, consisting of multiparous women with no previous CD, ≥37 weeks, who went into labor spontaneously. In that specific group, 22.4% underwent a CD, which raises some questions. Although this is lower than 38.2%, the group CD rate previously from the national tertiary center in Freetown.^36^ This is considerably higher compared with reports from other settings such as Pakistan (1.6%), Surinam (9.9%) and Tanzania (13.9%), and similar to Brazil (19.3%).^37^, ^38^, ^39^, ^40^ Women in the TGSC group 3 had delivered vaginally before, had no additional obstetric risk factors and therefore were expected to have a low CD rate. A possible explanation for the fact that almost a quarter of women in this group underwent a CD is the concentration of complicated cases in this group due to delays before arriving in the district and regional hospitals and untreated maternal morbidity such as hypertension and diabetes. Nevertheless, it can be reasonably argued that the implementation of Robson classification‐based clinical team audit cycles, combined with protocols enforcing stricter indications for CDs, can effectively reduce unnecessary CDs in this Robson group 3.^41^ Additionally, targeted team training in assisted vaginal births and improved availability and proficiency with vacuum equipment are critical for this approach.^38^, ^42^, ^43^, ^44^, ^45^ These interventions specifically address the modifiable causes of high CD rates, such as prolonged labor and the variation in clinical practice, thereby promoting safer and higher quality of obstetric care.

### 4.3 Perinatal mortality

The overall perinatal mortality of 7.6% and more specifically in the CD group with 9.9% reveals a great concern. Information on the viability of the fetus was not routinely recorded in records, which limits the interpretation of the data. High perinatal mortality after CD in Sierra Leone has been previously described.^36^, ^46^ High perinatal mortality is linked to the barriers that women face in reaching and receiving emergency obstetric care.^46^, ^47^ A perinatal mortality in the CD groups of above 20% was observed in group 4 (multiparous women without a previous CD, single cephalic, >37 weeks, induced or CD before labor) and 6 (nulliparous breeches). This could possibly be explained due to poor fetal monitoring, based on limited human resources and equipment. Another reason could be limited availability and adherence to induction and breech delivery protocols. Our data did not include registration of fetal heart rate before CD; this reflects the perilous situation many women and their babies are subjected too. Improved fetal monitoring could likely improve perinatal outcomes in this setting.^46^ Two groups (group 6, nulliparous breeches, and 7, multiparous breeches) with the highest recorded perinatal mortality were in the vaginal breech‐delivery group. Breech delivery is a team effort that demands a high level of obstetric skill. Given the complexity of such births, perinatal outcomes can be significantly improved through team‐based scenario training in breech delivery, with the active involvement of a senior professional—preferably a consultant—for hands‐on guidance.^48^ The AVD rate was low, showing potential opportunity for decreasing unnecessary second stage CDs by on‐the‐job training in the art of instrumental deliveries.^49^, ^50^ A previous study on vacuum extractions in Sierra Leone reported a lack of training and a lack of functional vacuum equipment as the two main barriers.^42^

### 4.4 Obstetric task‐sharing

In Sierra Leone, CDs are mostly performed in rural areas (61.7%), performed in public facilities (81.0%) and by ACs (57.7%).^27^ Even though ACs have gone through a rigorous 3‐year training program, with 1 year of housemanship, before being allowed to share obstetric tasks with MDs, safeguarding quality is a prerequisite.^51^ In a previous cohort‐study in Sierra Leone, non‐inferiority for maternal mortality was found for CDs performed by ACs compared with those performed by MDs.^28^ However, it is not only about the outcome, CDs also need to be clinically justifiable. This study identified variations in the indications for CDs between Acs and MDs. ACs performed a higher proportion of CDs for previous CD, malpresentation, and twin pregnancies, whereas MDs more frequently conducted CDs for fetal distress, uterine rupture, and breech presentation. These findings suggest that MDs are more involved in CDs with more complicated indications which is in line with previous research.^28^ Proper task‐sharing requires that more complex cases are handled by the most experienced and educated health providers, and this might explain the differences in indications and TGCS groups between ACs and MDs. Shared team responsibilities, non‐hierarchical collaboration and regular auditing are crucial components to provide optimal obstetric care.

Proper implementation of protocols requires teamwork and a collaborative attitude. This is where scenario training with the whole obstetric team can be of great value. In a task‐sharing context, the focus must always be on delivering the best possible care for mother and child—not on individual interests, but on teamwork, with a safe and collaborative approach free from ego, with a shared commitment to quality and safety.

### 4.5 Strengths and limitations

The project included all regional hospitals and five of the eight most productive district hospitals and therefore provides a good overview of the women delivering in governmental hospitals outside of the capital area. Data collection was completed in two rounds to minimize the need for exclusion of patients and to provide the most realistic picture. Presenting the deliveries that could not be classified in the TGCS provided the most realistic presentation.

The present study relies on retrospective data collection from maternity and theater registries and patient files, which may be subject to incomplete and inaccurate documentation. Information on induction of labor and the viability of the fetus were not consistently documented, which makes the interpretation of the findings for specific groups less accurate. In addition, only government hospitals outside of Freetown were included, and therefore findings cannot be generalized to private hospitals and hospitals in the area around the capital. Findings from the TGCS in the tertiary center in the capital have also been recently described.^36^

## 5 CONCLUSIONS

In the district and regional hospitals in Sierra Leone, the facility CD rate is high. High CD rates are also reported within the low a priori risk groups TGCS 1 (nulliparous women, single cephalic, ≥37 weeks, spontaneous labor) and 3 (multiparous women without a previous CD, single cephalic, ≥37 weeks, in spontaneous labor). In addition, the total AVD rate is low compared with other similar settings, suggesting that more effort is needed to reduce unnecessary CDs while safeguarding the access to necessary CDs and lowering the maternal and perinatal mortality. Almost half of the CDs are performed by ACs. Perinatal mortality is high, particularly for breech deliveries, suggesting need for hands on training and a call for a review of the training. Significant differences in the indications and the TGCS groups between ACs and MDs are reported. Further insight is required into the factors influencing decision making for CDs in this specific context.

## AUTHOR CONTRIBUTIONS

JW conceived the initial study objective and designed and planned with feedback and input from MR, RHL, HB and AJvD. Data collection was performed by GS, SAB, SS and JWu, supervised by JW. JW and AJvD performed the analysis. JW wrote the manuscript with input from all authors. All authors approved the final version of the manuscript.

## FUNDING INFORMATION

This study received funding from The Liaison Committee between the Central Norway Regional Health Authority and the Norwegian University of Science and Technology (NTNU) grant 90531700 and Laerdal Foundation for Acute Medicine, Grant/Award Number: 40495. JW, AJvD and HAB were supported by the Liaison Committee between the Central Norway Regional Health Authority and the Norwegian University of Science and Technology.

## CONFLICT OF INTEREST STATEMENT

JW, AJvD and HAB are unpaid Board Members of CapaCare, the NGO organizing the Surgical Training Program for MDs and ACs in Sierra Leone in collaboration with the Ministry of Health. SS has received allowances for conducting data collection.

## Supporting information


**Table S1.** Robson Ten‐Group Classification Report Table.
**Figure S1.** Deliveries per hospital by Robson Ten‐Group Classification.

## Data Availability

Data are stored securely and they are available upon request.
