# Supplementary material for: A cross‐sectional study of caesarean deliveries performed by medical doctors and associate clinicians in nine district and regional hospitals in Sierra Leone using the Robson Ten Group Classification System
Source: Int J Gynaecol Obstet. 2026 May 30;175(2):865–76. doi: 10.1002/ijgo.71072 (PMC13674845; doi:10.1002/ijgo.71072)
Supplement: Supplementary file 1 [file IJGO-175-865-s001.docx]

**Supplementary Table 1. Robson Ten-Group Classification Report Table**

| Robson TGCS group | | No. of CD | Total in group | Relative size of the group | Group CD rate | Absolute contribution of group to overall CD | Relative contribution of group CD to overall CD |
| --- | --- | --- | --- | --- | --- | --- | --- |
| 1 |  | 450 | 1656 | 28.8% | 27.2% | 7.8% | 25.2% |
| 2 |  | 22 | 29 | 0.5% | 75.9% | 0.4% | 1.2% |
| 3 |  | 604 | 2702 | 47.0% | 22.4% | 10.5% | 33.8% |
| 4 |  | 63 | 71 | 1.2% | 88.7% | 1.1% | 3.5% |
| 5 |  | 190 | 291 | 5.1% | 65.3% | 3.3% | 10.6% |
| 6 |  | 35 | 66 | 1.1% | 53.0% | 0.6% | 2.0% |
| 7 |  | 59 | 109 | 1.9% | 54.1% | 1.0% | 3.3% |
| 8 |  | 134 | 281 | 4.9% | 47.7% | 2.3% | 7.5% |
| 9 |  | 42 | 45 | 0.8% | 93.3% | 0.7% | 2.4% |
| 10 |  | 141 | 445 | 7.7% | 31.7% | 2.5% | 7.9% |
| Missing | | 46 | 53 | 0.9% | 86.8% | 0.8% | 2.6% |
| Total | | 1786 | 5748 | 100.0% | 31.1% | 31.1% | 100.0% |

CD = Caesarean delivery, TGCS = Ten Group Classification System. Groups: 1 = Nulliparous, single cephalic, ≥37 weeks, spontaneous labour; 2 = Nulliparous, single cephalic, ≥37 weeks, induced or CD before labour; 3 = Multiparous (excluding previous CD), single cephalic, ≥37 weeks, in spontaneous labour; 4 = Multiparous (excluding previous CD), single cephalic, >37 weeks, induced or CD before labour; 5 = Previous CD, single cephalic, ≥ 37 weeks; 6 = nulliparous breeches; 7 = Multiparous breeches; 8 = Multiple pregnancies; 9 = Abnormal lies; 10 = Single cephalic, <37 weeks.

**Supplementary Figure 1: Deliveries per hospital by Robson Ten-Group Classification**

|  | Group 1 | Group 2 | Group 3 | Group 4 | Group 5 | Group 6 | Group 7 | Group 8 | Group 9 | Group 10 |
| --- | --- | --- | --- | --- | --- | --- | --- | --- | --- | --- |
| Regional hospital 4 | 57 | 2 | 101 | 2 | 14 | 6 | 5 | 16 | 3 | 19 |
| Regional hospital 3 | 266 | 0 | 503 | 5 | 33 | 11 | 5 | 35 | 8 | 52 |
| Regional hospital 2 | 291 | 1 | 358 | 2 | 27 | 13 | 20 | 33 | 4 | 69 |
| Regional hospital 1 | 384 | 8 | 470 | 4 | 78 | 9 | 26 | 41 | 8 | 79 |
| District hospital 5 | 55 | 2 | 169 | 7 | 38 | 1 | 7 | 22 | 1 | 33 |
| District hospital 4 | 148 | 0 | 303 | 6 | 25 | 8 | 12 | 39 | 4 | 57 |
| District hospital 3 | 180 | 13 | 385 | 33 | 47 | 8 | 11 | 43 | 4 | 56 |
| District hospital 2 | 117 | 3 | 170 | 9 | 12 | 2 | 10 | 25 | 8 | 39 |
| District hospital 1 | 158 | 0 | 243 | 3 | 17 | 8 | 13 | 27 | 5 | 41 |

| Robson  Group |  |  |  |  |  |  |  |  |  |  |
| --- | --- | --- | --- | --- | --- | --- | --- | --- | --- | --- |

Robson Ten Group Classification System groups: 1 = Nulliparous, single cephalic, ≥37 weeks, spontaneous labour; 2 = Nulliparous, single cephalic, ≥37 weeks, induced or CD before labour; 3 = Multiparous (excluding previous CD), single cephalic, ≥37 weeks, in spontaneous labour; 4 = Multiparous (excluding previous CD), single cephalic, >37 weeks, induced or CD before labour; 5 = Previous CD, single cephalic, ≥ 37 weeks; 6 = nulliparous breeches; 7 = Multiparous breeches; 8 = Multiple pregnancies; 9 = Abnormal lies; 10 = Single cephalic, <37 weeks.
